# Volume addiction: introducing a novel phenotype within compulsive high volume eating

**DOI:** 10.3389/fpubh.2025.1760037

**Published:** 2026-01-12

**Authors:** Vera I. Tarman

**Affiliations:** University of Toronto Temerty Faculty of Medicine, Toronto, ON, Canada

**Keywords:** binge eating disorder, compulsive high volume eating, eating addiction, food addiction, volume addiction, eating disorder, ultra-processed food addiction

## Abstract

Most accounts of disordered eating and food addiction focus on hedonic, calorie-dense intake. However, many patients describe an urgent drive to feel profoundly full, sometimes achieved through enormous volumes of low-calorie foods or fluids. This presentation centers on amount rather than food type, and differs from binge eating disorder's 2-h consumption criterion. Current diagnostic systems (DSM, ICD) do not recognize this volume-seeking presentation, and scientific literature rarely treats gastric volume as a primary reinforcer. Water-load studies show individuals with bulimia nervosa and binge-eating disorder consume substantially greater volumes before reporting satiation and display abnormal gastric myoelectrical activity compared to controls. Animal studies demonstrate that gastric stretch receptors and vagal responses are plastic and can alter satiety and intake. Gut hormones, such as ghrelin, leptin, insulin, and GLP-1s, and their interactions with reward circuitry link visceral signals to feeding behavior. Neuroimaging studies reveal altered reward responses to food cues and volume. Together, these findings suggest volume itself is a meaningful, measurable dimension warranting clinical and scientific attention. This article synthesizes these strands to propose compulsive high-volume eating (CHVE) as a distinct construct, defined by recurrent, distressing episodes where gastric distension serves as the central motivator. Within CHVE, I propose a “volume-addiction” subtype characterized by craving, tolerance, and loss of control focused on fullness. While current evidence is indirect, sufficient data exists to justify identification and study of this phenotype to target research and develop more effective treatments for food-addicted individuals who struggle specifically with volume intake.

## Introduction

Discussions of disordered eating and food addiction typically center on overconsumption of calorie-dense, highly palatable foods. In these narratives, the core pathology lies in the hedonic and metabolic value of nutrients: sugar, fat, and energy-dense combinations that strongly engage reward circuitry (1).

Volume addiction presents differently from classic binge eating. Rather than pursuing flavor and immediate gratification followed by guilt and remorse as is defined by binge eating disorder (2), individuals seek the physical sensation of gastric distension itself, using food merely as a vehicle for achieving stomach tightness and pressure. Someone might consume enormous quantities of vegetables, clear broth, sugar-free gelatin, or plain water, all in the attempt to maximize volume while minimizing calories. The drive isn't flavor or hedonic pleasure; it's the physical sensation of distension itself, even to the point of being uncomfortably overfull.

This pattern raises a straightforward but underexplored question: can gastric distension itself function as a primary reinforcer for a subset of individuals, giving rise to a recognizable high-volume compulsive eating phenotype and perhaps even to a form of “addiction” to fullness? Existing DSM diagnostic categories such as BED and BN do not distinguish between those who binge predominantly for taste and calories and those who binge, at least in part, to attain a specific interoceptive state of extreme fullness (3). Research cohorts have been recruited based on binge frequency and total energy intake rather than fullness-seeking behavior as the primary driver. Consequently, individuals whose compulsion centers on achieving distension through relatively low-calorie, high-volume intake may be systematically misclassified or overlooked entirely.

This is the reason why despite mounting clinical observations and physiological evidence supporting the existence of volume addiction, its true prevalence remains unknown because this specific pattern of compulsive, fullness-seeking eating has never been distinctly measured or systematically assessed in research studies. Yet clinicians working with eating disorders and food addiction consistently encounter this presentation in their practices, making it a recognizable and recurring clinical phenomenon even in the absence of formal epidemiological data. There is an urgent need for targeted studies and diagnostic criteria that can accurately capture and quantify this under-recognized clinical phenotype.

Fortunately, recent advances in neuro-gastroenterology provide a framework for understanding the proposed construct of “volume addiction.” We now know that stretch-sensing nerve endings in the stomach wall communicate directly with brain reward centers through the vagal nerve and that gut hormones, such as GLP-1, ghrelin and peptide YY, amongst others, modulate both homeostatic energy balance and mesolimbic reward pathways, thus creating a bridge between physical stomach filling and emotional satisfaction (4–6). Critically, from the addiction framework, these pathways show remarkable plasticity. They adapt in response to dietary composition, chronic stress, and dietary patterns in ways that may explain tolerance development (7–9).

Indeed, from an addiction-model perspective, compulsive high-volume eating (CHVE) at its extreme maps remarkably onto substance use disorder criteria: cue-triggered craving for fullness sensations (similar to drug craving), progressive escalation to overcome tolerance (needing larger volumes over time), and persistent difficulty controlling intake despite clear negative consequences such as social withdrawal, physical pain, medical complications. Viewing CHVE through an addictive lens opens therapeutic possibilities. Interventions proved effective in substance use disorders, from specific medication to behavioral strategies addressing cravings and triggers, could potentially be adapted for volume-driven eating, though systematic research has barely begun.

This article synthesizes the available literature on voluminous eating and gastric signaling, on hormonal biology and neuroimaging, to develop an argument for two related constructs. The first is compulsive high-volume eating (CHVE), in which recurrent episodes of very large intake are motivated substantially by pursuit of gastric distension. The second is a narrower volume-addiction subtype of CHVE, in which the sensation of fullness exhibits addiction-like properties including craving, tolerance, and continued use despite harm. This article concludes by emphasizing the significance of this concept, which aims to guide research and improve treatment options for individuals with food addiction, especially those who struggle with excessive food consumption.

## The neurobiology of volume-driven eating: gastric stretch receptors and vagal signaling

Understanding CHVE begins with the dynamic communication between stomach and brain. Eating triggers specialized stretch receptors embedded in the stomach wall, which send signals via the vagal nerve to brain regions governing satiety and emotion (10). This vagal pathway activates the parasympathetic “rest and digest” response, fostering physiological calm. Many people intuitively use stomach fullness to trigger this relaxation response when stressed, but repeated overuse can dysregulate this feedback loop.

Animal studies show gastric mechanoreceptors show striking plasticity: diets high in fat, chronic psychological stress, and circadian disruption can all blunt receptor sensitivity, making greater stomach expansion necessary to feel comparably full (7–9). This sets the stage for a cycle where an increasing amount of volume is needed to achieve comfort. This is a hallmark of tolerance. Over time, frequent overstretching acts like wearing out a rubber band: natural signals of fullness fade, and people may eat far beyond normal limits to recapture fleeting satisfaction. Chronic stress further distorts these cues, while irregular sleep can erode the daily rhythm of gastric sensitivity, promoting disordered intake around the clock. Losing the reliable sense of satiation as feedback is central to compulsive high-volume eating and can fuel the urge to pursue fullness at any cost.

## Hormonal dysregulation: leptin, ghrelin, and insulin

Beyond mechanical pathways, hormones are deeply implicated in volume addiction. Leptin, released by fat stores and a stretching stomach, normally signals satiety. But in people with weight gain or ongoing large-volume intake, it has been shown that leptin resistance develops. This translates to the brain becoming numb to leptin's fullness message, requiring even more stretch or fat mass to prompt satiety (11). This adaptive resistance means the threshold for stopping eating keeps climbing.

Elevated ghrelin compounds the problem. Ghrelin, the hunger-signaling hormone produced when the stomach is empty, should decrease as the stomach fills during a meal high in volume. However, in individuals with obesity or eating disorders, ghrelin levels may remain chronically elevated. When ghrelin stays high, people continue feeling hungry despite gastric fullness, pushing them to consume beyond their body's actual requirements (12). This persistent hunger drive caused by a blunted ghrelin response, coupled with leptin resistance, creates a powerful biological urge to consume large volumes even in the absence of a true energy deficit.

Insulin resistance further disrupts appetite control. Beyond managing blood sugar, insulin works as a crucial appetite-control messenger, quieting hunger signals in conjunction with leptin and other satiety hormones (13). When insulin resistance develops, this signaling pathway becomes disrupted, potentially contributing to increased hunger and overeating. Blood sugar spikes and crashes drive intense cravings even when the stomach is distended with low-calorie foods that don't address the underlying glucose deficit.

Gut hormones such as GLP-1 and Peptide YY modulate both homeostatic energy balance and mesolimbic reward pathways, creating a biological bridge between mechanical stomach filling and emotional satisfaction. GLP-1 receptors are expressed not only in hypothalamic satiety centers but also throughout the mesolimbic reward system. When the stomach distends, GLP-1 simultaneously triggers homeostatic satiety while dampening dopamine-mediated reward signaling. Chronic overstretching may theoretically blunt both pathways, requiring progressively larger volumes to achieve relief, thus driving the compulsive pursuit of extreme gastric distension (14, 15).

As tolerance escalates, individuals move from eating for comfort and reward to eating to escape discomfort, mirroring the addiction cycle seen in substance use. Smaller meals no longer satisfy; only maximal stretch produces relief. Skipping or reducing volume, in contrast, can provoke negative emotions, physical unease, and a feeling of “emptiness”; this describes the withdrawal-like symptoms of classical addiction theory. This progressive blunting and cycle of relief-seeking are defining features of the volume addiction phenotype.

## Habit, conditioning, and tolerance

The brain's reward system adds another layer of complexity. Each time we experience pleasurable meals, the brain links dopamine surges to eating contexts such as celebrations, special occasions, or specific feelings of fullness. This “euphoric recall” persists and encourages overeating even after food loses its appeal. Someone might chase the intense fullness remembered from a memorable holiday dinner and eat past comfortable satiety. As with substance addiction, repeated exposure leads to desensitization, with neuro-adaption in the reward and stress circuits close behind: these are classic features of addiction (16). As tolerance and dependence grow, even everyday life can be shaped by the pursuit and challenges of maintaining or controlling volume intake: social gatherings, travel, or unpredictable schedules become sources of anxiety if they threaten access to “enough” volume. This relentless cycle increases the risk not only of medical complications but also of social isolation and distress.

## Human evidence: water load tests and gastric function

The most compelling human evidence that tolerance to fullness can develop comes from water load test (WLT) paradigms combined with electrogastrography (EGG), which measures the stomach's electrical activity coordinating gastric contractions. In these protocols, participants drink water at a steady pace until reaching satiation and maximal fullness; their volume is measured and compared across groups. van Dyck et al. (4) examined women with BN and BED using two-step WLT with EGG recording. Compared with healthy controls, BN/BED participants ingested significantly larger water volumes before reporting satiation and maximal fullness, and their EGG recordings revealed lower gastric myoelectrical activity after water ingestion. Critically, this electrical dysfunction correlated with the number of binge episodes, in that those with more severe binges showed greater gastric electrical abnormalities. The findings of elevated volume tolerance plus correlated electrical dysfunction provide objective biological evidence that repeated high-volume intake produces measurable changes in how the stomach functions.

Notably, participants in this and other studies supporting these findings (17, 18) were recruited based on standard BN/BED criteria rather than by primary distension-seeking complaints that might not meet BN/BED criteria, and laboratory water intake may not capture the phenomenology of spontaneous, compulsive high-volume episodes with low-calorie foods in real life. But it supports the concept that the feeling of fullness can be influenced by repeated stimulation of the stretch receptors.

## Diagnostic distinction from classical binge eating

Although research increasingly points to the reality of compulsive high-volume eating, no studies to date have formally set CHVE apart from conventional binge eating disorder (BED). Existing diagnostic systems remain anchored in caloric abundance, loss of control, and emotional aftermath and have typically focused on those experiencing rapid consumption of highly palatable foods (3). In contrast, the CHVE/volume addiction subtype is defined by craving for fullness, relative indifference to taste and timeline (grazing being a prominent feature of this subgroup) and persistence despite adverse consequences or lack of pleasure. These features parallel those of substance addiction.

While water load studies provide objective evidence of altered volume tolerance, clinical identification of volume addiction requires attention to patient-reported motives and behaviors that distinguish it from classical binge eating. Clinically, individuals with volume addiction may describe eating large quantities of deliberately chosen low-calorie foods, such as raw vegetables, air-popped popcorn, sugar-free gelatin, or plain rice cakes, explicitly to achieve stomach distension rather than taste satisfaction. Patients often report that “feeling stuffed” provides relief, calm, or a sense of completion. Unlike typical binge eating, where palatability drives consumption, these individuals may express indifference to food quality, stating “it could be anything. I just need the full feeling.”

Withdrawal-like symptoms emerge during restriction attempts, including intrusive thoughts about eating, anxiety focused on stomach emptiness, irritability until fullness is restored. Medical complications may include electrolyte disturbances from excessive fluid intake, gastric overdistension, particularly dangerous in post-bariatric surgery patients, and bowel obstruction from fiber-rich bulk foods. Despite awareness of these risks and associated distress, the compulsion to pursue fullness persists, paralleling the continued-use-despite-harm criterion central to addiction diagnosis (19, 20). Given that the literature on eating disorders frequently details these health problems, is it possible that volume addiction is either a component of or wrongly categorized as an eating disorder? We urgently need research to differentiate between these two groups.

## Treatment implications: behavioral and biological approaches

Volume addiction presents a unique treatment challenge. Traditional models that focus mainly on emotional triggers or restricting “forbidden foods” often do not reach patients whose principal compulsion is mechanical stretch, not taste or calories. For these individuals, behavioral approaches need to restore reliability to internal cues, often through rigorous external structure. This might involve weighing, measuring, and planning portions of food, even at the expense of social ease (21). Flexible but consistent rules can help counter both under- and overeating when internal regulation has collapsed.

Deep brain stimulation, which targets reward and impulse control networks in the nucleus accumbens, could theoretically be adapted to treat fullness-seeking as a compulsive reward-driven behavior, though this remains untested for volume addiction (22).

The Vibrating Ingestible BioElectronic Stimulator (VIBES) represents another unique approach originally developed for obesity treatment. This ingestible capsule delivers vibrational stimulation to gastric mechanoreceptors, creating “illusory distension” that triggers hormonal responses such as increased serotonin and GLP-1 without actual stomach filling. In swine models, VIBES reduced food intake by 40%. Because VIBES directly targets mechanosensory pathways hypothesized to drive volume addiction, it could theoretically satisfy stretch-seeking drives without requiring massive food or fluid intake, potentially preventing medical complications of extreme volume consumption (23, 24).

For individuals with CHVE and volume addiction, whose gastric interoceptive sensitivity is demonstrably altered (as evidenced by elevated water load tolerance and abnormal gastric myoelectrical activity), gastric biofeedback could serve as a targeted behavioral intervention. Tiemann et al. recently showed that gastric biofeedback training using electrogastrography (EGG) can improve satiation sensing, fullness perception, body listening, and intuitive eating (25). By enhancing awareness of gastric stretch signals and recalibrating the threshold at which fullness is perceived, this approach may reduce the compulsive drive to pursue extreme distension. Combined with structured cognitive-behavioral therapy, gastric biofeedback could help patients learn to recognize and trust moderate fullness cues, distinguish comfortable from harmful distension, and gradually shift away from volume-seeking behaviors (26). The scalability and accessibility of 2D biofeedback make it particularly promising for both clinical settings and home-based intervention programs.

GLP-1 receptor agonists represent a promising avenue, reducing intake and binge frequency through combined effects on gastric emptying, postprandial satiety, and dampening mesolimbic responses to food cues. However, clinical experience suggests that after 6 months to a year, intrusive thoughts about food typically resurface despite continued weight loss effectiveness, creating a situation where patients feel driven toward larger portions yet face severe digestive consequences (27).

## Clinical recognition and public health implications

Volume addiction matters for several critical reasons. First, it can occur even with healthy foods. Someone attempting to curb intake of indulgent foods may turn to carrots or celery or water, only to discover their addictive impulses have shifted from flavors to sheer volume of any ingestible item. This becomes particularly dangerous for individuals who've undergone weight loss surgery or take GLP-1 medications, risking complications like bowel obstruction or stomach rupture. Bariatric surgery patients face heightened vulnerability: frequent small meals create repeated fullness sensations that may strengthen addiction pathways, potentially serving as a gateway to volume-based compulsion (28–30).

Second, acknowledging the biological underpinnings could reduce shame and blame. Patients who compulsively eat past fullness often face embarrassment and humiliation. If clinicians frame this as a condition rooted in brain chemistry and hormonal dysregulation, rather than poor willpower or gluttony, it opens doors to more compassionate and effective interventions (31). This understanding might make patients more receptive to exploring therapeutic approaches that could help them tremendously.

## Research mandate: bridging gaps to validate volume addiction

Three critical deficiencies call for investigation to establish volume addiction as an empirically validated syndrome:

**1. No prospective cohort definition**

The volume-driven CHVE phenotype has not been systematically identified or characterized. Thus, a research priority would be to develop screening tools that distinguish fullness-seeking from palatability-seeking motives, then prospectively recruit participants across diverse clinical settings using standardized assessments of volume tolerance, craving for distension, and withdrawal patterns during restriction attempts.

**2. Absence of mechanistic biomarkers**

Despite evidence that gastric stretch pathways, hormonal systems, and reward circuits interact to drive compulsive eating, no studies have profiled these systems specifically in volume-motivated individuals. Conducting water load tests with concurrent EGG, gastric accommodation imaging, and serial hormone measurements (leptin, ghrelin, and GLP-1) could help determine if volume addiction has distinct physiological signs compared to hedonic binge eating.

**3. No targeted treatment trials**

Promising interventions such as GLP-1 agonists, gastric biofeedback, mechanosensory devices (VIBES), and addiction-model behavioral therapies could be tested for volume-specific compulsion. Conduct early-phase trials incorporating objective endpoints (water load tolerance, EGG normalization, fullness craving scales, study of relapse to high-volume episodes) to determine whether mechanistic interventions targeting stretch-reward pathways outperform other more standard BED protocols in volume-driven patients.

Closing these gaps will transform volume addiction from a clinical observation into a testable construct with validated diagnostic criteria, biological markers, and evidence-based treatment.

## Conclusion

The defining trait of volume addiction, a phenotype of clinical importance that's frequently overlooked, is the need to eat large quantities of food to feel full, rather than focusing on calories or taste. Numerous studies have revealed a relationship between this eating behavior and shifts in satiety signals, hormonal irregularities, and reward mechanisms within the body. Together these changes can produce tolerance and withdrawal-like symptoms, consistent with addiction models. Clinical and research frameworks have enough data to adopt this perspective so that diagnosis and treatment can focus on fullness-seeking mechanisms rather than caloric intake alone. If we can fill the current research voids with precise cohort definitions, mechanism investigations, and tailored interventions, the idea of volume addiction will shift from just a description to a tangible, scientifically supported syndrome. This transition forecasts more effective diagnoses, customized therapeutic interventions, and more successful results for people grappling with overeating, particularly those at the intersection of neurobiology, eating disorders, and food addiction.

## Data Availability

The original contributions presented in the study are included in the article/supplementary material, further inquiries can be directed to the corresponding author.
